# *Rotylenchus wimbii* n. sp. (Nematoda: Hoplolaimidae) associated with finger millet in Kenya

**DOI:** 10.21307/jofnem-2021-016

**Published:** 2021-02-22

**Authors:** Phougeishangbam Rolish Singh, Gerrit Karssen, Kelvin Gitau, Cecilia Wanjau, Marjolein Couvreur, Njira Njira Pili, Godelieve Gheysen, Wim Bert

**Affiliations:** 1 Nematology Research Unit, Department of Biology, Ghent University, K.L. Ledeganckstraat 35, 9000, Ghent, Belgium; 2 National Plant Protection Organization, Wageningen Nematode Collection, P.O. Box 9102, 6700, HC Wageningen, The Netherlands; 3 Meru University of Science and Technology, P.O. Box 972-60200, Meru, Kenya; 4 Department of Biological Sciences, Moi University, P.O Box 3900-30100, Eldoret, Kenya; 5 Department of Biotechnology, Ghent University, Coupure Links 653, 9000 Gent, Belgium

**Keywords:** 18S, 28S, *COI*, ITS, Finger millet, Kenya, Morphometrics, New species, Phylogeny, Plant-parasitic nematodes, *Rotylenchus*, SEM, Systematics, Taxonomy

## Abstract

*Rotylenchus wimbii* n. sp. was found associated with finger millet in Kenya and is described based on light microscopy, scanning electron microscopy, and molecular information. Sequence analysis was performed on ITS, 18S, and D2-D3 of 28S of ribosomal DNA and *COI* of mitochondrial DNA. This new species is characterized by a moderate female body size of 0.6 to 0.8 mm, a continuous hemispherical lip region with four annuli, 3 to 4 irregular blocks on the basal lip annule, absence of longitudinal cuticular striations in anterior region, four lateral lines forming three equal bands which are areolated mainly at pharynx level, a robust stylet of 23 to 27 µm of which 45 to 53% is cone part, and with rounded to sometimes indented knobs, a secretory-excretory pore around level of pharyngo-intestinal junction, didelphic-amphidelphic reproductive system, vulva without distinct epiptygma, indistinct to empty spermatheca, tail usually truncated with 5 to 9 annuli, phasmids located at 7 to 17 annuli anterior to anus, and absence of males. Molecular phylogenies, in combination with species delimitation, supported the distinctiveness of *Rotylenchus wimbii* n. sp. and revealed some mislabeled *Rotylenchus brevicaudatus* sequences in GenBank.

Within the plant-parasitic nematode (PPN) genus *Rotylenchus* Filipjev, 1936 (Nematoda: Hoplolaimidae), 104 valid species have been recognized (Nguyen et al., 2019). *Rotylenchus* spp. have been reported from all the continents, being found associated with numerous important crops ranging from rice and cereals to tubers, vegetables, and even ornamental plants and trees. As obligate root parasites feeding on root hairs or epidermal or cortical cells of a plant, they can cause prominent damage to the host plant, with symptoms ranging from stunted and reduced growth to wilting, chlorosis, reduced root system and root lesions, often leading to significant losses in crop yield (Castillo and Vovlas, 2005; Manzanilla López and Marbán Mendoza, 2012; Sikora et al., 2018). To facilitate the identification of *Rotylenchus* species, Castillo and Vovlas (2005) have developed a tabular or matrix key using a total of 11 characters of this nematode group. This identification system has so far been widely accepted and its widespread use has also recently been facilitated by a web-based key that draws its basis from cluster analysis (Nguyen et al., 2019). In combination with the morphological features, molecular information such as ITS, 18S, and 28S of ribosomal DNA and *COI* of mitochondrial DNA have been used for their identification.

In the current paper, we characterize a newly discovered *Rotylenchus wimbii* n. sp. found associated with finger millet, Eleusine coracana (L.) Gaertn. (Planta: Poaceae) in Kenya. The potential importance of this nematode as a pest of finger millet was evidenced by its dense population in the soil sample and its presence observed in the finger millet root system (revealed by the fuchsin staining method). Nematode characterization was carried out based on morphological information obtained from light microscopy (LM) and scanning electron microscopy (SEM) studies. Illustrations, morphometrics, and molecular information of ITS, 18S, and 28S of rDNA and *COI* of mtDNA are also provided for the novel species.

## Materials and methods

### Sample collection and nematode extraction

A soil sample mixed with some roots was collected using a shovel from 15 to 25 cm soil depth in a zig-zag pattern from a finger millet field [Eleusine coracana (L.) Gaertn.] in Kipkaren Estate, Eldoret, Kenya in February, 2019. The GPS coordinates of the field location are 00°30.395’ N; 035°14.825’E. Nematodes were extracted from 100 ml of soil by using modified Baermann’s method (Whitehead and Hemming, 1965). The extracted nematodes were stored at 4°C during the course of analysis.

### Root staining

Host roots were stained using acid fuchsin (Byrd et al., 1983). For this, clean roots were bleached in 2.5% NaOCl for 5 min, followed by rinsing the roots in running tap water, and boiling them in 30 ml distilled water with 1 ml of stock staining solution (0.35 g acid fuchsin, 25 ml acetic acid, and 75 ml distilled water) in a microwave for 30 sec. After cooling, excess fuchsin was drained and roots were washed with running tap water and de-stained by immersing them in 70% acidified glycerol, and finally observed under a stereo microscope for presence of stained PPN.

### Morphological characterization

For morphological studies, live nematodes were heat-relaxed by quickly passing over a flame in a drop of water on a glass slide until nematode movement stopped and examined, photographed, and measured using an Olympus BX51 DIC Microscope (Olympus Optical, Tokyo, Japan), equipped with an Olympus C5060Wz camera as described in Singh et al. (2018). After recording morphological information, each specimen was recovered from the slide and its genomic DNA was extracted. For fixing, the nematode suspension obtained after extraction was concentrated in a drop of water in a glass embryo dish, followed by addition of a few drops of Trump’s fixative [2% paraformaldehyde, 2.5% glutaraldehyde in 0.1 M Sorenson buffer (Sodium phosphate buffer at pH = 7.5)] into it. The nematodes were then immediately heated in a microwave (700 watts) for about 4 sec and left for 1 hr at room temperature and finally at 4°C for 24 hr. This was followed by gradually transferring the nematodes to anhydrous glycerin, ready to be mounted on glass slides as described in Singh et al. (2018). For SEM, specimens fixed in Trump’s fixative were washed in 0.1 M phosphate buffer (pH = 7.5) and dehydrated in a graded series of ethanol solutions, critical-point-dried with liquid CO_2_, mounted on stubs with carbon tabs (double conductive tapes), coated with 25 nm gold, and photographed with a JSM-840 EM (JEOL) at 12 kV (Singh et al., 2018).

### Molecular characterization

After morphological analysis, heat-relaxed nematodes were recovered from temporary slides and each individual nematode was cut into pieces in distilled water using a blade and the pieces were transferred to a PCR tube containing 20 µl of worm lysis buffer [50 mM KCl, 10 mM Tris at pH = 8.3, 2.5 mM MgCl_2_, 0.45% NP 40 (Tergitol Sigma), 0.45% Tween 20]. The PCR tubes were incubated at −20°C (10 min) followed by addition of 1 µl proteinase K (1.2 mg/ml), incubation at 65°C (1 hr) and 95°C (10 min), and finally centrifuging the mixture at 14,000 rpm for 1 min (Singh et al., 2018). PCR amplifications of the partial ITS, 18S, and D2-D3 expansion segment of 28S of rDNA were done using the primer pairs, Vrain2F: 5´-CTTTGTACACACCGCCCGTCGCT-3´/Vrain2R: 5´-TTTCACTCGCCGTTACTAAGGGAATC-3´ (Vrain et al., 1992), SSU18A: 5´-AAAGATTAAGCCATGCATG-3´/SSU26R: 5´-CATTCTTGGCAAATGCTTTCG-3´ (Mayer et al., 2007) and D2A: 5´-ACAAGTACCGTGAGGGAAAGTTG-3´/D3B: 5´-TCCTCGGAAGGAACCAGCTACTA-3´ (Nunn, 1992), respectively, using thermal profiles described in Singh et al. (2018, 2019). For amplification of the *COI* region of mtDNA, the primer pair, JB3: 5´-TTTTTTGGGCATCCTGAGGTTTAT-3´/JB4.5: 5´-TAAAGAAAGAACATAATGAAAATG-3´ was used according to Bowles et al. (1992). The PCR products were enzymatically cleaned as mentioned in Singh et al. (2020) and contigs were made from the newly produced forward and backward sequences using Geneious Prime 2020.0.5 (https://www.geneious.com) and deposited in GenBank.

### Phylogenetic analysis

The phylogenetic relationships of the new species with other related species were analyzed based on the partial sequences of ITS, 18S, 28S, and *COI*. Phylogenetic programs implemented in Geneious Prime 2020.0.5 were used. The obtained consensus contigs were subjected to BLAST search to check for closely related species on GenBank and all the collected sequences for each gene fragment were aligned using MUSCLE alignment of Geneious Prime 2020.0.5 using default parameters, followed by manually trimming of the poorly aligned ends. The best nucleotide substitution model of each gene alignment was determined by jModelTest 2.1.10 and using the selected models, phylogenetic trees were created using Bayesian phylogenetic analyses (MrBayes 3.2.6), which was run under 1 × 10^6^ generations (4 runs) and Markov chains sampled at every 100 generations, and 20% of the converged runs regarded as burn-in (Huelsenbeck and Ronquist, 2001). To check for additional distinctiveness of our sequences in phylogenetic trees, the species delimitation plugin of Geneious Prime 2020.0.5 (Masters et al., 2011) was used to calculate Rosenberg’s PAB, which tests the probability for reciprocal monophyly of sequence clusters (Rosenberg, 2007).

## Results

### Systematics

*Rotylenchus wimbii* n. sp.

**Figure 1:**
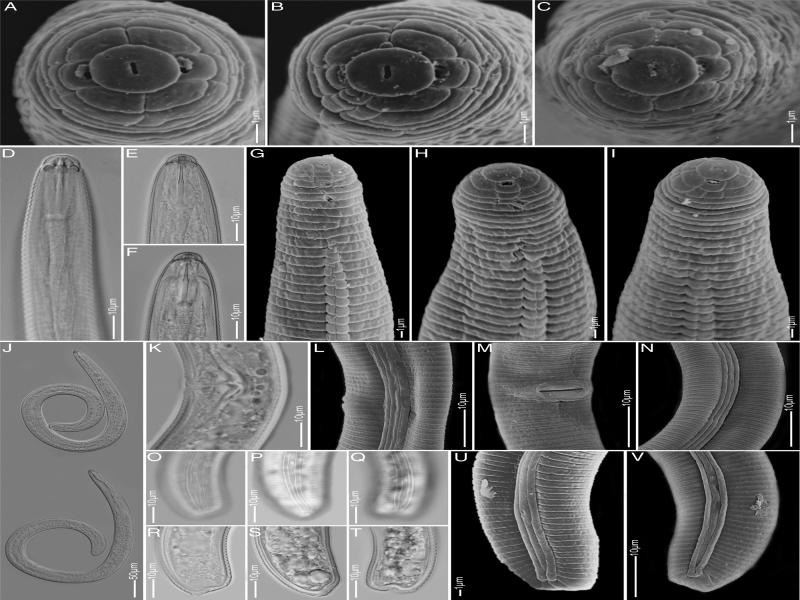
Light microscopy and scanning electron microscopy images of *Rotylenchus wimbii* n. sp. female. A to C: *En face* view; D to I: Anterior part of the body showing lip and neck region; J: Whole female body; K to N: Vulva region; O to V: Tail region.

**Figure 2:**
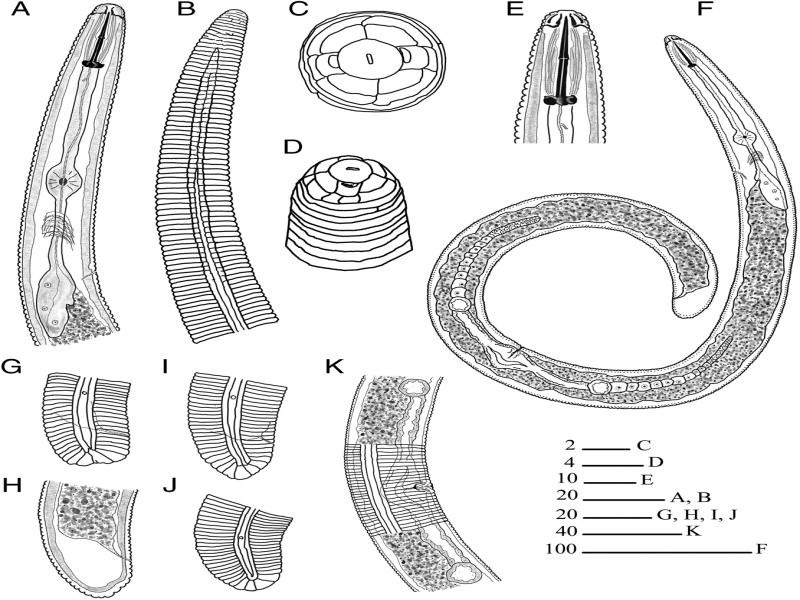
Illustrations of *Rotylenchus wimbii* n. sp. female. A, B: Anterior part of the body showing lip and neck region; C: *En face* view; D, E: Lip region; F: Whole body; G to J: Tail region; K: Vulva region. Scales are given in µm.

Figures 1 and 2, Tables 1 and 2.

**Table 1.** Morphometric data for fixed female *Rotylenchus wimbii* n. sp. specimens mounted in glycerin.

Female character	
*n*	19
Body length (*L*)	690 ± 44 (620-760)
*a* = *L*/MBD	27.3 ± 3.0 (23.0-33.0)
*b* = *L*/Anterior end to pharynx-intestine junction	5.8 ± 0.3 (5.0-6.0)
*b′* = *L*/Anterior end to end of pharyngeal gland	5.1 ± 0.4 (4.5-6.1)
*c* = *L*/Tail length	66.0 ± 12.0 (49.0-91.0)
*c′* = Tail length/ABD	0.6 ± 0.1 (0.3-0.8)
*V* = Anterior end to vulva opening/*L* × 100	58 ± 1 (55-60)
Lip region height	4.6 ± 0.3 (4.2-5.3)
Lip region width	8.2 ± 0.3 (7.7-8.7)
Stylet length	25.3 ± 0.9 (22.5-26.6)
Cone length	12.5 ± 0.7 (10.8-13.7)
Shaft length	10.3 ± 0.5 (9.4-11.2)
Stylet knob height	2.6 ± 0.4 (1.9-3.4)
Cone % Stylet	49.3 ± 1.8 (44.8-52.6)
Dorsal gland opening from stylet base	5.4 ± 0.6 (4.1-6.3)
Anterior end to secretory-excretory pore	107 ± 6.2 (93.2-118)
Anterior end to pharynx-intestine junction	118 ± 5.3 (105-128)
Anterior end to pharyngeal gland end	135 ± 6.0 (121-144)
Anterior end to vulva opening	400 ± 24.8 (341-408)
Maximum body diameter (MBD)	25.5 ± 2.9 (21.5-31.7)
Anal body diameter (ABD)	16.9 ± 1.4 (15.2-19.9)
Tail length	10.8 ± 2.1 (6.9-13.4)
Phasmid to tail tip length	31.6 ± 3.1 (25.1-36.3)
Number of annuli between phasmid and tail tip	20 ± 3 (12-26)
Number of tail annuli	7 ± 1 (5-9)

**Note:** All measurements, except ratios and V%, are in µm and in the format: mean ± standard deviation (range).

**Table 2.** Comparison of some important female characters and information on availability of males of 23 *Rotylenchus* species reported from Africa.

Species	Body length (mm)	Lip region	Lip annuli number	Stylet length (µm)	Cone% of stylet	Spermatheca in matured females	V%	Male	Tail annuli number	Phasmid position
*R. abnormecaudatus*	0.6-0.7	Broadly rounded, setoff to continuous	4-5	24-25	42-45	Not seen	56-59	Not found	8-11	8-14 annuli anterior to anus
*R. acuspicaudatus*	0.8-1.0	Broadly rounded, slightly setoff	4-5	26-28	43-51	Rounded, filled with sperms	54-57	Found	13-16	7-15 annuli anterior to anus
*R. alius*	0.5-0.9	Truncate, blunt anterior, not setoff	4-5	20-31	42-51	Rounded, filled with sperms	54-66	Found	8-15	3-4 annuli posterior to anus
*R. bialaebursus*	0.6-0.8	Rounded, not setoff	4	26-29	46-50	Rounded, filled with sperms	54-59	Found	6-8	9-12 annuli anterior to anus
*R. brevicaudatus*	0.5-0.8	Broad, rounded, not setoff	4-5	18-26	–	Present	52-62	Found	5-11	8-14 annuli anterior to anus
*R. capensis*	0.7-1.2	Hemispherical, slightly setoff	5-7	25-35	39-45	Rounded, filled with sperms	54-59	Found	9-14	1-3 annuli anterior to anus
*R. catharinae*	0.8-1.1	Hemispherical, slightly setoff	7-8	30-33	45-48	Rounded, filled with sperms	55-56	Found	13-14	1-4 annuli anterior to anus
*R. caudaphasmidius*	0.6-0.9	Hemispherical, not setoff	5	24-32	–	Rounded, filled with sperms	55-63	Found	12	5 annuli posterior to anus
*R. cypriensis*	0.5-0.8	Hemispherical, well setoff	3-4	21-28	–	Small and empty	57-67	Not found	6-7	7-14 annuli anterior to anus
*R. devonensis*	0.7-0.9	Conoid, blunt, not setoff	4	25-34	43-46	Rounded, filled with sperms	53-64	Found	7-9	8-14 annuli anterior to anus
***R. wimbii*** **n. sp**.	**0.6-0.8**	**Hemispherical, not setoff**	**4**	**23-27**	**45-53**	**Indistinct to empty**	**55-60**	**Not found**	**5-9**	**7-17 annuli anterior to anus**
*R. gracilidens*	0.9-1.2	Hemispherical, slightly setoff	4	29-35	–	Rounded, filled with sperms	53-59	Found	8-12	15-20 annuli anterior to anus
*R. incultus*	0.6-1.0	Hemispherical, not setoff	4-5	22-32	–	Developed, filled with sperms	53-61	Found	ca 8	13-14 annuli anterior to anus
*R. karooensis*	0.7-1.0	Rounded, slightly setoff	4-5	21-27	43-47	Rounded, filled with sperms	51-60	Found	7-15	1-6 annuli anterior to anus
*R. kenti*	0.5-0.7	Rounded, not setoff	4-5	21-25	44-52	Rounded, filled with sperms	60-66	Found	9-17	3-4 annuli anterior to anus
*R. mabelei*	0.9-1.1	Hemispherical, slightly setoff	4-5	27-30	43-50	Empty	48-57	Not found	14-18	3 annuli anterior to 5 annuli posterior of anus
*R. minutus*	0.5-0.8	Angularly hemispherical, slightly setoff	3-4	20-27	56-58	Rounded, filled with sperms	53-66	Found	9-14	1 annule anterior to 4 annuli posterior of anus
*R. mirus*	0.6-0.7	Hemispherical, setoff	4-5	20-23	43-50	Rounded, filled with sperms	55-58	Found	6-10	5-12 annuli anterior to anus
*R. robustus*	0.9-1.6	Hemispherical, well setoff	6-7	33-50	50-56	Rounded, filled with sperms	52-58	Found	8-17	7 annuli anterior to 3 annuli posterior of anus
*R. rugatocuticulatus*	1.0-1.3	Rounded, slightly setoff	4	35-42	–	Developed, filled with sperms	51-56	Found	12	8-10 annuli anterior to anus
*R. triannulatus*	0.6-0.8	Rounded, well setoff	3	24-25	43-48	Rounded, filled with sperms	56-60	Found	10-11	Anal level to 8 annuli anterior
*R. unisexus*	0.5-0.9	Hemispherical, not setoff	4	20-29	–	Not seen	47-59	Not found	9-13	6-7 annuli anterior to anus
*R. usitatus*	1.1-1.2	Flattened anteriorly, not setoff	4-5	29-29	43-47	Rounded, filled with sperms	45-55	Found	10-16	8-14 annuli anterior to anus

**Note:** The new species is given in bold.

### Description

#### Female

Body moderately large (0.6-0.8 mm), habitus spiral to 6-shaped when heat relaxed. Lateral field differentiation starting as single areolated band, gradually forming two areolated bands up to level of metacorpus valve and further continuing as three areolated bands up to isthmus level, after which bands becomes smooth till tail terminus. Longitudinal cuticular striations in anterior region absent. Labial region hemispherical with 4 annuli, not offset from body but appears to have a slight depression under LM. *En face* showing rounded labial disc, marked from rest of labial region, not elevated, slit like oral aperture, lateral sectors with two amphidial apertures and smaller than subdorsal and subventral sectors. Basal lip annule with 2 to 3 longitudinal striations (from six face views) forming 3 to 4 irregular blocks, but in one specimen with five striations forming six irregular blocks. Stylet robust with large, rounded, and sometimes indented knobs. Dorsal pharyngeal gland opening at 4 to 6 µm from stylet base. Pharynx with well-developed median bulb, valves, slender isthmus, and gland overlapping intestine by maximum 30 µm dorsally. Secretory-excretory pore often at level of pharyngo-intestinal junction. Hemizonid distinct, just above secretory-excretory pore, about two body annuli long. Reproductive tract didelphic-amphidelphic, each branch equally developed with outstretched ovaries containing rows of developing eggs, vulva at 55 to 60% of body length from anterior end, without distinct epiptygma. Spermatheca indistinct to rounded, not filled. Tail very short with commonly truncated tip or sometimes slightly hemispherical to rounded tip. Phasmid pore like, 7 to 17 annuli anterior to anus.

#### Male

Not found.

### Diagnosis and relationships

*Rotylenchus wimbii* n. sp. is characterized by a moderate body size of 0.6 to 0.8 mm, a hemispherical, continuous lip region with four annuli, basal lip annule with 3 to 4 irregular blocks, absence of longitudinal cuticular striations at anterior region, lateral field with four lines forming three equal bands which are areolated around pharynx level, a robust stylet of less than 30 µm of which 45 to 53% is cone and with rounded to sometimes indented knobs, didelphic-amphidelphic reproductive system, vulva without distinct epiptygma, indistinct to empty spermatheca, very short truncated tail with 5 to 9 annuli, and pore like phasmids located at 7 to 17 annuli anterior to anus. According to Castillo and Vovlas (2005), the matrix code of this species is A4, B1, C1, D4, E1, F2, G3, H (commonly truncated), I2, J2, K1.

This new species is morphologically closest to Rotylenchus abnormecaudatus Van den Berg and Heyns, 1974 and *Rotylenchus brevicaudatus *Colbran, 1962. The females of these three species have similar body sizes, lip region of more or less hemispherical to rounded and very slightly offset to continuous with 4 to 5 annuli, stylet length below 30 µm, lateral field areolation only at pharynx level, absence of longitudinal cuticular striations, vulva position of 52 to 62% of body length from anterior end, phasmids positioned at 7 to 17 annuli anterior to anus, and short tail with only 5 to 11 tail annuli. The new species differs from *R. abnormecaudatus* in having 4 vs 4 to 5 lip annuli, a slightly longer cone part of stylet (45-53% vs 42-45%), and often truncated vs irregularly rounded tail terminus. *Rotylenchus abnormecaudatus* has also been reported with a peculiar and irregular arrangement of annuli and lateral field ending in the posterior part of tail which is not seen in other species including *R. wimbii* n. sp. While males of *R. wimbii* n. sp. and *R. abnormecaudatus* are not known and subsequently, no sperm is observed in the female spermathecae, males of *R. brevicaudatus* have been reported and the female spermatheca are often filled with sperm. *Rotylenchus wimbii* n. sp. further differs from *R. brevicaudatus* by the presence of 2 to 3 longitudinal striations on the basal lip annule vs 6 to 12 such longitudinal striations as reported by Sher (1965) from the study of six face views of the 20 female topotypes supplied by R.C. Colbran, and commonly truncated vs hemispherical tail terminus. Information on the number of longitudinal striations on basal lip annule for *R. abnormecaudatus* is not available.

The new species is also comparable to Rotylenchus cypriensis Antoniou, 1981, *Rotylenchus mabelei* Van den Berg and De Waele, 1989, and *Rotylenchus unisexus* Sher, 1965, all of which are species without males and reported to occur in South Africa and Kenya. The new species can be separated from *R. cypriensis* in continuous vs well offset lip region and absence vs presence of a ventral mucron at the tail tip. It can be differentiated from *R. mabelei* by a slightly smaller body (0.6-0.8 vs 0.9-1.1 mm), a lesser number of tail annuli (5-7 vs 14-18), and the position of phasmids with respect to anus (7-17 anterior vs 3 anterior to 5 posterior of anus). Finally, our new species can be differentiated from *R. unisexus* by the longitudinal striae on basal lip annule (3-4 vs 16-18 striae), tail annuli number (5-9 vs 9-13), and phasmid position (7-17 annuli anterior vs 6-7 annuli anterior of anus). An additional comparison of the new species with female morphological characters and morphometrics of all the *Rotylenchus* species that have been reported from Africa till date is provided in Table 2.

### Etymology

The species epithet refers to its host. Wimbi originates from Swahili and is used as a common name for finger millet in Eastern Africa.

### Type host and locality

The new species was found parasitizing the host plant *Eleusine coracana* (L.) Gaertn. (Planta: Poaceae), commonly known as finger millet, Kipkaren Estate, Eldoret, Kenya. The host status was also confirmed by a positive greenhouse test at the National Plant Protection Organization, Wageningen, The Netherlands. The GPS coordinates of the type location are 00°30.395’ N; 035°14.825’E.

### Type material

Female holotype (Slide: WT 3796) and seven female paratypes in two slides (Slides: WT 3797 and WT 3798) are deposited at the National Plant Protection Organization, Wageningen Nematode Collection (WaNeCo), the Netherlands. One slide containing 10 female paratypes is deposited at Ghent University Museum, Zoology Collections, Belgium. An additional slide containing eight female paratypes is also deposited at UGent Nematode Collection (Slide: UGnem-300) of Nematology Research Unit of Ghent University, Belgium.

### Root staining

Several stained spiral-shaped nematodes were observed in the host roots which are *Rotylenchus*. As no other *Rotylenchus* species or other spiral PPN such as *Scutellonema* and *Helicotylenchus* was detected from the corresponding soil sample, the PPN observed in the roots are most likely *R. wimbii* n. sp. confirming that this novel species can parasitize finger millet.

### Molecular characterization

#### D2-D3 of 28S rDNA

**Figure 3:**
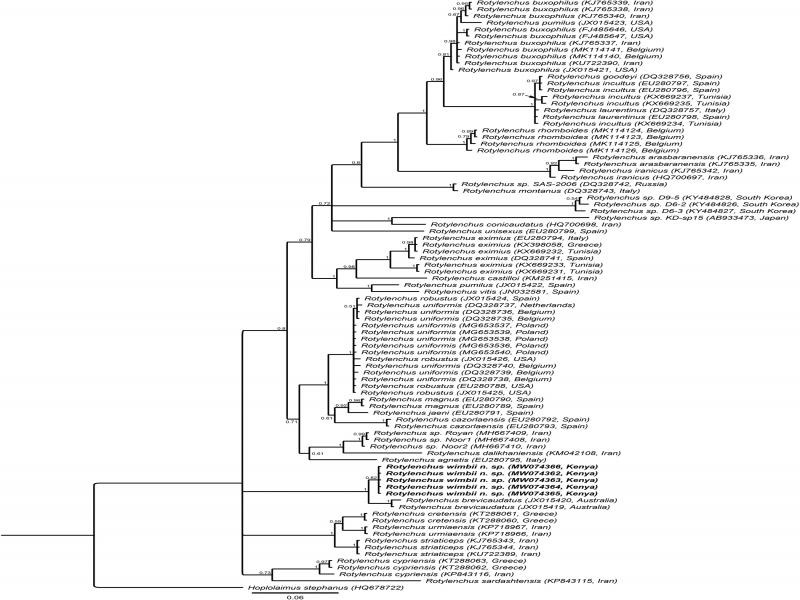
BI phylogenetic tree generated from the analysis of D2-D3 of 28S rDNA sequences using GTR + G + I nucleotide substitution model. Bayesian posterior probabilities are given next to each node and sequences of *Rotylenchus wimbii* n. sp. are in bold.

Five sequences of up to 770 bp without any sequence variation were produced (MW074362-MW074366). They were found to be most similar to a sequence of *R. brevicaudatus* (JX015419) with 98.5% similarity (11 differences in 740 bp). These five sequences were aligned with 74 identified *Rotylenchus* sequences (28 species) and eight unidentified *Rotylenchus* sequences. The phylogenetic tree (Fig. 3) inferred revealed a maximally supported sister relationship of *R. wimbii* n. sp. with two *R. brevicaudatus* sequences (JX015419 and JX015420) originating from Australia, and both species appeared to be distinct according to species delimitation (significant Rosenberg’s PAB: 0.02). However, the clade containing these two species has unresolved position with respect to other *Rotylenchus* species. Two partial 28S sequences of *R. brevicaudatus* from South Africa (MN262454 and MN262456) are found 18.2% (139 bp) different from each other and about 20% (150 bp) different from our 28S sequences. However, these South African sequences are not linked to any morphological data (Lamula, 2020), have a remarkable intra-population variation (18%), have poor alignment with other sequences, and are therefore, most likely mislabeled.

#### 18S of rDNA

Six sequences of up to 903 bp without sequence variation were produced (MW074378-MW074383). The closest *Rotylenchus* sequence available from the databank was a *R. unisexus* sequence (MK809263) with 98.9% similarity (9 differences in 810 bp). No phylogenetic tree was made using 18S due to limited availability of 18S sequences of *Rotylenchus* from GenBank.

#### ITS of rDNA

**Figure 4:**
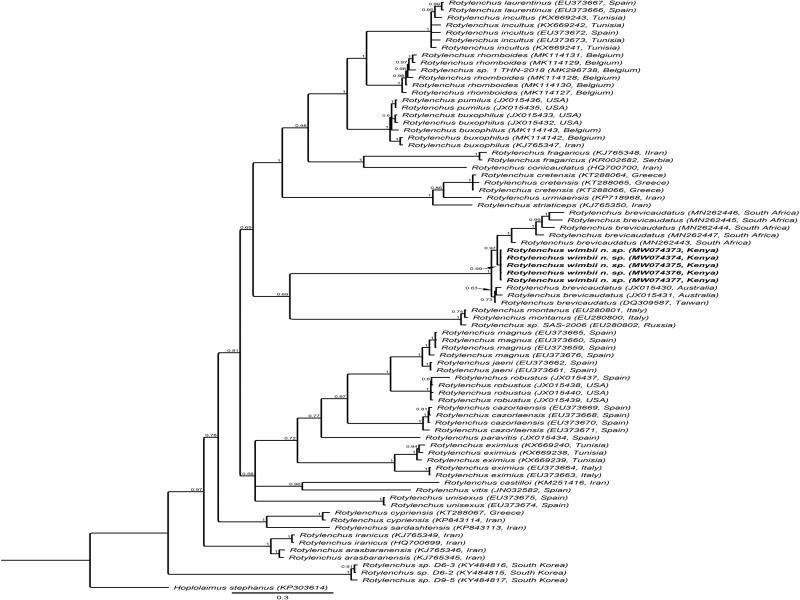
BI phylogenetic tree generated from the analysis of ITS of rDNA sequences using GTR + G + I nucleotide substitution model. Bayesian posterior probabilities are given next to each node and sequences of *Rotylenchus wimbii* n. sp. are in bold.

Five sequences of up to 973 bp without sequence variation were produced (MW074373-MW074377) which were found closest to a *R. brevicaudatus* (DQ309587) sequence from Taiwan with 96.9% similarity (25 differences in 818 bp). These sequences were aligned with 68 identified (25 species) and five unidentified *Rotylenchus* sequences. The resulting phylogenetic tree (Fig. 4) seemingly inferred our sequences within a maximally supported *R. brevicaudatus* clade, consisting of populations of South Africa, Australia, and Taiwan. However, the ITS sequences (MN262443-MN262447) of South Africa with a remarkable intra-population variation of 8 to 14% (71-141 bp) are not linked to morphological data of the associated species (Lamula, 2020; see also 28S), and are distinctive from *R. wimbii* n. sp. (significant Rosenberg’s PAB: 8.8E^−4^) as well as from the Australian-Taiwanese *R. brevicaudatus* clade (also significant Rosenberg’s PAB: 8.8E^−4^), and are, therefore, most likely mislabeled. Our sequences of *R. wimbii* n. sp. were also found distinct from the Australian-Taiwanese *R. brevicaudatus* sequences (significant Rosenberg’s PAB: 5.8E^−4^) which are linked with morphological data and, therefore, do most likely represent the genuine *R. brevicaudatus* sequences (Cantalapiedra-Navarrete et al., 2013). Thus, ITS analysis also supports *R. wimbii* n. sp. as a distinct species.

#### COI of mtDNA

**Figure 5:**
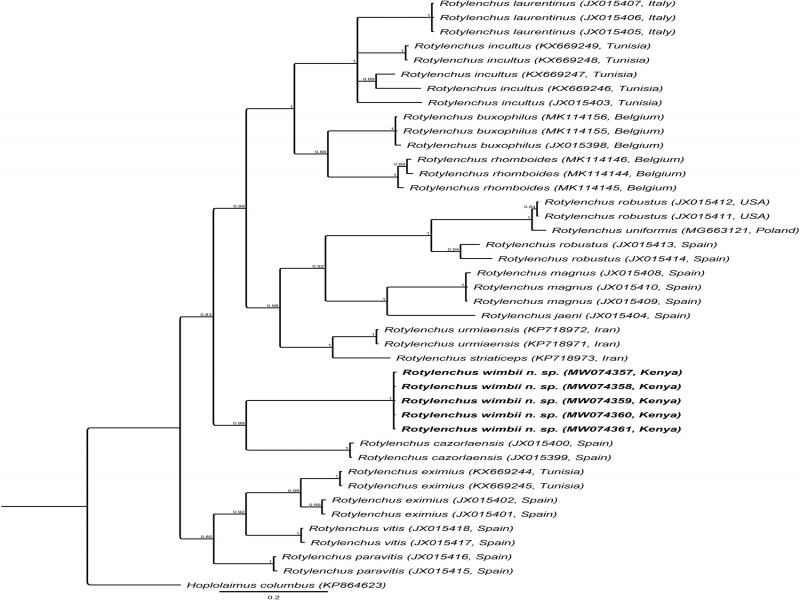
BI phylogenetic tree generated from the analysis of *COI* of mtDNA sequences using GTR + G + I nucleotide substitution model. Bayesian posterior probabilities are given next to each node and sequences of *Rotylenchus wimbii* n. sp. are in bold.

Five sequences of up to 414 bp, without any sequence variation, were produced (MW074357-MW074361) and the closest *Rotylenchus* sequence was Rotylenchus urmiaensis Noruzi et al., 2015 (KP718972) with 80.73% similarity (79 differences in 410 bp). These sequences were aligned with 36 identified sequences from 14 *Rotylenchus* species. The phylogenetic tree (Fig. 5) inferred revealed a moderately supported (PP = 0.89) clade of the new species with *Rotylenchus cazorlaensis* Castillo and Gómez Barcina, 1987 sequences (JX015399 and JX015400) from Spain.

## Discussion

A total of 15 *Rotylenchus* species have been originally described from Africa, i.e. *R. abnormecaudatus*, *Rotylenchus acuspicaudatus* Van den Berg and Heyns, 1974, *Rotylenchus alius* Van den Berg, 1986a, *Rotylenchus bialaebursus* Van den Berg and Heyns, 1974, *Rotylenchus capensis* Van den Berg and Heyns, 1974, *Rotylenchus catharinae* Van den Berg and Heyns, 1974, *Rotylenchus devonensis* Van den Berg, 1976, *Rotylenchus incultus* Sher, 1965, *Rotylenchus karooensis* Van den Berg, 1986a, *Rotylenchus kenti* Van den Berg, 1989, *R. mabelei*, *Rotylenchus mirus* Van den Berg, 1986b, *Rotylenchus triannulatus* Van den Berg and Heyns, 1974, *R. unisexus*, and *Rotylenchus usitatus* Van den Berg and Heyns, 1974. Except for *R. incultus* and *R. unisexus*, which were described from crops in Zimbabwe and Kenya respectively, all other species were described from studies in South Africa indicating the relatively unexplored species diversity of *Rotylenchus* in the African continent. A few other *Rotylenchus* species such as *R. brevicaudatus*, *Rotylenchus caudaphasmidius* Sher, 1965, *R. cypriensis*, *Rotylenchus minutus* (Sher, 1964) Germani, Baldwin, Bell & Wu, 1985, *Rotylenchus robustus* (de Man, 1876) Filipjev, 1936 and *Rotylenchus rugatocuticulatus* Sher, 1965 were also reported from samples sourced in South Africa (Ali et al., 1973, Van den Berg, 1976, 1978, 1981, 1986a, b, 1989, Van den Berg and Heyns, 1974; Zeidan and Geraert, 1989).

Morphologically, the new species is easily separated from all the African species, except for *R. brevicaudatus* for which the absence of males and the number of longitudinal striations on the basal lip annule and tail terminus are crucial. The longitudinal striations are visible under LM and have been already illustrated in the work of Sher (1965), but the inclusion of SEM analysis has highly improved the usability of this character in our species delimitation. Revealing subtle morphological differences using SEM is especially useful for species with overlapping morphometrics and morphology, such as here with *Rotylenchus* spp. The small species differences observed in the morphology are also clearly visible in the molecular analyses, including molecular species delimitation, based on the sequences of ITS, 18S, D2-D3 of 28S of rDNA and *COI* of mtDNA. This study also revealed that the ITS and D2-D3 ‘*R. brevicaudatus*’ sequences originating from South Africa, which are not linked to morphological data, have most likely been mislabeled. This finding highlights the importance of an unequivocal link between morphology and DNA sequences in order to prevent a cascade of sequence-based misidentifications (Janssen et al., 2017).

The quantitative and qualitative impact of this newly discovered parasite of Kenyan finger millet remains to be studied, so as to assess the level of crop losses caused by the pest. This is especially important in the context of finger millet as it represents an emerging cereal crop of potentially great importance in the tropical and the sub-tropical regions.
